# Effects of Dietary Taurine on Maturation Indices, Antioxidant Capacity, Ovaries Amino and Fatty Acids Profile, and Vitellogenin Gene Transcription Level in *Penaeus vannamei* Female Brooders

**DOI:** 10.1155/2024/5532545

**Published:** 2024-11-05

**Authors:** Mansour Torfi Mozanzadeh, Mahmoud Nafisi Bahabadi, Vahid Morshedi, Amin Oujifard, Naser Agh, Ahmad Ghasemi, Khalegh Maneii, Hadi Ebrahimi, Shirin Hamedi, Rezvan Tamadoni

**Affiliations:** ^1^South Iran Aquaculture Research Centre, Iranian Fisheries Science Research Institute (IFSRI), Agricultural Research Education and Extension (AREEO), Ahwaz, Iran; ^2^Faculty of Nano and Bio Science and Technology, Persian Gulf University, Bushehr, Iran; ^3^Persian Gulf Research Institute, Persian Gulf University, Bushehr, Iran; ^4^Artemia and Aquaculture Research Institute, Urmia University, Urmia, Iran

**Keywords:** lipid metabolism, maturation, penaeid shrimp, taurine, vitellogenin

## Abstract

A 30-day research was carried out to examine the impacts of dietary taurine (Tau) on ovaries maturation and physiological responses of *Penaeus vannamei* female brooders (29.4 ± 0.2 g). A basal diet (497 g kg^−1^ protein and 140 g kg^−1^ lipid) was administered with graded levels of Tau ranging from 0 (control) to 2, 4, 6, 8, and 10 g kg^−1^. A total of 180 shrimp brooders were stocked into 18 250 L black circular polyethylene tanks. Female (*n* = 5) and male (*n* = 5) shrimps were stocked in each tank and supplied with seawater (35.2 ± 3.1 g L^−1^ salinity, 28.9 ± 1.4°C) and the experimental feeds were offered to shrimp twice a day at 5% of their biomass. Supplementing diet with 4–8 g Tau kg^−1^ reduced latency period after eye stalk ablation to spawning (5–6 days) that was associated with higher hepatopancreatic and gonadosomatic (except for 8 g Tau kg^−1^ diet) indices (*p* < 0.05). With 10 g Tau kg^−1^ diet hepatopancreas glutathione peroxidase activity and total antioxidant capacity increased and catalase activity increased by 6 g Tau kg^−1^ diet. Supplementing diet with Tau-enhanced bile-salt dependent lipase activity in the gut. Docosahexaenoic acid and Tau levels were elevated in the ovaries with the increment of dietary Tau level (*p* < 0.05). Plasma total protein, calcium, cholesterol, and high-density lipoprotein increased with inclusion of 6–10 g Tau kg^−1^ diet. The transcription levels of *vitellogenin*, *insulin-like growth factor II*, *superoxide dismutase*, *prophenoloxidase*, and *lysozyme* genes transcription levels were upregulated in the hepatopancreas of shrimp brooders fed 6–10 g Tau kg^−1^ diet (*p* < 0.05). It seems that Tau at 4–8 g kg^−1^ diet by modulating lipid metabolism, antioxidant capacity, and immunocompetence can improve maturation and health status of *P. vannamei* brooders.

## 1. Introduction

The whiteleg shrimp *Penaeus vannamei* with 6.8 million tons aquaculture production is the most farmed aquatic species in the world [1]. This euryhaline species can tolerate a wide range of culture condition (e.g., temperature and salinity), low protein and low fishmeal (FM) content diet, and has rapid growth rate [2]. Many studies are carried out regarding the nutritional requirements of this species during adult and maturation stages to promote its spawning quality and more research are required to elevate its reproduction efficiency and offspring quality. In a review study on the nutritional requirements of Penaeid brooders, Wouters et al. [3] reported that dietary protein level in artificial formulated feeds should range between 45% and 55% that usually are cofed with a fresh food mixture. Regarding essential amino acids (AAs), 10 AAs, including arginine, histidine, isoleucine, leucine, lysine, methionine, phenylalanine, threonine, tryptophan, and valine, are considered as essential AA (EAA) [4], whereas tyrosine and cysteine are determined as semi-EAA for most crustaceans [5]. AAs increase the ovarian maturity development and enhance the reproductive performance by yolk proteins, polypeptide hormones, and enzymes biosynthesis [3]. For instance, dietary supplementation of arginine, the main precursor of nitric oxide, which is a potent bioactive messenger in crustacean, surged ecdysterone level in the Chinese mitten crab (*Eriocheir sinensis*) [6]. Furthermore, tryptophan is the main precursors of 5-hydroxytryptamine and has a vital role in polyamines regulation [7] that are essential for steroidogenesis and follicular development and ovulation [8]. However, information regarding other EAA, semiessential or conditionally EAA such as taurine (Tau) on reproductive performance of crustaceans are scarce.

Tau (2-aminoethanesulfonic acid) is a conditionally essential sulfonic AA for farmed aquatic species based on their life stage, culture condition, health status, dietary biochemical composition, and feed ingredients [9]. This AA is a main component of bile salts (i.e., taurocholic acid and taurochenodeoxycholic acid) that have key role in lipid digestion [10]. Tau is a potent reactive oxygen species (ROS) scavenger and have antioxidant characteristics [11]. In addition, several research proved that Tau can alleviate negative effects of high plant protein content diets on performance of cultured aquatic species [12–14]. In this regard, Yue et al. [15] reported supplementing high plant protein content diet with Tau-enhanced *P. vannamei* growth. A study in *P. monodon* has shown the Tau synthesize capacity in *Penaeid* shrimp, but this ability is markedly can be affected by the dietary cystine level [16]. In this regard, Richard et al. [16] reported that supplementing methionine-deficient diets (30%–50% deficiency in methionine) with cystine not only increased daily protein accretion but also significantly enhanced hemolymph Tau level in black tiger shrimp (*P. monodon*) fed 30% methionine deficient diet. Thus, it seems that adjusting dietary cystine levels could serve as an alternative approach to meet dietary Tau requirements in shrimp. Previous studies in various crustacean species also confirmed that Tau can improve growth, feed efficiency, antioxidant capacity, immune responses, disease resistance, and stress tolerance during larvae and juvenile stages [15, 17–21]. In *P. monodon*, dietary Tau requirement is about 4 g kg^−1^ [22] meanwhile in *P. vannamei*, supplementing diet with 2 g Tau kg^−1^ did not improve growth [17].

Recent research on some fish species showed that supplementing diet with Tau markedly improved reproductive performance such as fecundity, fertilization and hatching rates, larval quality, and survival rate [23–26]. It is suggested that dietary Tau can trigger vitellogenesis, increase Tau concentration in the eggs AA pool, protect germ cells from oxidative stress, and elevate the effects of sex steroids on the gametogenesis [23–28]. Therefore, in our research, it was determined to examine the influence of dietary Tau on some reproductive factors and physiological responses of *P. vannamei* female brooders.

## 2. Materials and Methods

### 2.1. Experimental Diets

A graded amount of Tau ranging from 0 (control), 2, 4, 6, 8, and 10 g kg^−1^ was added to six isonitrogenous (497 g kg^−1^ protein) and isolipidic (140 g kg^−1^ lipid) diets (Table 1). According to findings from earlier research [30, 31], dietary energy was formulated to be *Ca*. 19.0 MJ kg^−1^ [29]. The dry ingredients were mixed with a hand mixer (Philips HR1560, Hungary) for 10 min, then a blend of fish oil, canola oil, and soy lecithin was added to the dry mixture and blended for another 10 min. Finally, enough distilled water was poured into the mixture and blended for 10 min to form a dough. The dough was then cold pelleted using a meat grinder (Pars Khazar Buffalo-2020, Iran) to create 3 mm pellets. After being dried at 50°C for 24 h in a convection oven (Kimia Teb Gostar DH160, Iran), the pellets were kept at −20°C up to use.

### 2.2. Husbandry Trial

Subadult shrimp were moved to Persian Gulf University's Aquatic Research Laboratory from a greenhouse pond (Delvar, Bushehr, Iran). As advised by Alday-Sanz [32], shrimp brooders were treated with formalin bath (100 ppm, 30 s). Shrimp brooders were acclimated to the husbandry condition for 2 weeks in two 4000 L circular fiberglass tanks and were fed with the control diet twice daily (1200 and 2300 h). A total of 180 shrimp brooders, with a mean ± standard error (SE) of 29.4 ± 0.2 g, were distributed into 18 250 L black circular polyethylene tanks. Each tank held five females and five males, resulting in a 1 : 1 ratio. Two hundred liters of UV-disinfected seawater was supplied for each tank and equipped with a 300 W aquarium heater to stabilize the temperature at 28.9 ± 1.4°C. The other physicochemical parameters of the water, such as salinity, dissolved oxygen, and pH were maintained at 35.2 ± 3.1 g L^−1^, 5.5 ± 0.2 mg L^−1^ and 8.2 ± 0.2, respectively. Water exchange rate was 80% daily. The shrimp brooders were subjected to a 12 light : 12 darkness artificial photoperiod. Because fresh foods (e.g., squid and blood worms) have high levels of Tau, they were not offered to brooders. The experimental diets twice a day (1200 and 2300 h) were offered to shrimps at 5% of their biomass [31]. The unfed pellets were syphoned an hour after each feeding.

### 2.3. Sampling

The shrimp's biometry was done at the beginning and the end of the experiment. After 30 days of the feeding, the female brooding specimens' left eyestalk was ablated by cutting the base of the eye peduncle, followed by burning the wound. To assess the latency period following eyestalk ablation (ESA), the development of the ovaries was monitored every day by flashlight beam into the dorsal surface of the exoskeleton to evaluate the gonads size and color [32]. After ESA female brooders reached their final maturation stage (IV). The shrimp (*n* = 3 per tank) were put in chilled water (4°C, 10 min) to reduce stress and first their hemolymph was taken, then their ovaries, hepatopancreas, and gut were dissected and weighed [31]. The ovaries were aliquoted into three parts (*Ca*. 300 mg each) to evaluate absolute fecundity, AA, and fatty acid (FA) profiles. Also, hepatopancreas was aliquoted into two parts (*Ca*. 400 mg each) to evaluate antioxidant and genes transcription level and all samples were kept at −80°C. The egg diameter (μm) was measured with a light microscope (×40 magnification) equipped with a micrometer. To determine absolute fecundity, ovaries tissue (*n* = 3 per tank) (cranial, mid, and caudal sections) were taken and preserved in Gilson's fluid for 2 months [33].

### 2.4. FA and AA Profiles

Lipid was extracted based on method described by Folch, Lees, and Sloane-Stanley [34] and FA methyl esters conducted according to Christie method [35]. The FA profile of samples was measured by a gas chromatography (GC, Agilent technologies 7890 N, USA), equipped with a flame ionization detector and a cyanopropyl–phenyl capillary column (DB-225 MS, 30 m × 0.250 mm ID × 0.25 μm film thickness, USA), as described by Agh, Jasour, and Noori [36].

Freeze-dried samples (Freeze dryer, Operon, OPRFDU 7012, Sought Korea) were hydrolyzed (HCl [6 N], 24 h, 110°C) in glass vials filled with nitrogen. The o-phthaldialdehyde was used as a precolumn derivatization reagent according to Lindroth and Mopper [37]. Total AA levels were determined by high-performance liquid chromatography (HPLC) (Knauer, Germany) using C18 column (Knauer, Germany) at the flow rate of 1 mL min^−1^ with fluorescence detector (RF-530, Knauer, Germany).

### 2.5. Antioxidant Status and Digestive Enzymes

First, hepatopancreas was washed with ice-cold sodium chloride (9 g L^−1^) and were homogenized (1 : 10, w/v) in ice-cold buffer (100 mM Tris-HCl, 0.1 mM ethylenediaminetetraacetic acid (EDTA), 0.1% (v/v) triton X-100, pH 7.8) then centrifuged (11,312 g, 30 min, 4°C) and the supernatants were extracted. Catalase (CAT) [38], superoxide dismutase (SOD) [39], and glutathione peroxidase (GPx) [40] were determined by standard methods. Total antioxidant capacity (TAC) was measured using TAC assay kit (Navand Salamat Company, Iran).

Samples were homogenized (10,000 *g*, ×60 s, 4°C) in Tris-HCl buffer (1 : 3 W : V) (50 mM, pH 7.5, 4°C) and centrifuged (10,000 *g*, 20 min, 4°C). The supernatant was separated and preserved at −80°C [41]. *α*-Amylase (AMYL) [42], bile-salt activated lipase (LIP) [43], and total protease (PRO) [44] activities were evaluated by standard methods. Bradford method [45] was used to determine total soluble proteins in the samples. A microplate reader (Biotek Synergy HT, USA) was used to measure the specific activity of all enzymes.

### 2.6. Biochemical Analyses

Hemolymph was obtained from the base of the pleopod at the first abdominal segment near the genital pore (*n* = 3 samples per replicate, *n* = 9 samples per diet), using a syringe containing 400 µL of precooled anticoagulant (10 mM Tris-HCl, 250 mM sucrose, 100 mM sodium citrate, pH 7.6, 4°C) and centrifuged (6000 *g*, 10 min, 4°C) and plasma separated and stored at −80°C. An autoanalyzer (Technicon RA-1000, Technicon Instruments, New York, NY, USA) used to quantify total protein (TP), calcium (Ca^2+^), total cholesterol (CHOL), high-density lipoprotein (HDL), and triglyceride (TRIG) with clinical diagnostic kits (Pars Azmoon Kit, Tehran, Iran).

### 2.7. Evaluation of Relative Genes Expression in the Hepatopancreas

Total RNA in hepatopancreas was isolated using a RNXTM kit (Cinnagen, Tehran, Iran) based on the manufacturer instructions. Primers of RT-qPCR and references for *β*-actin, *vitelogenin* (*vtg*), *insulin-like growth factor II* (*IGF-II*), *SOD*, *prophenoloxidase (ProPO)*, and l*ysozyme (LYZ)* mRNA were showed in Table 2. In this assay, the *β*-actin was used as reference gene. Real-time PCR was executed by using Maxima SYBR Green/ROX qPCR Master Mix (Thermo Fisher Scientific, USA) based on the manufacturer's instructions and 1 μg cDNA. The results were evaluated in real-time PCR (CFX Connect Real-Time PCR Detection System, USA). Triplicate amplification reactions were carried out for each sample. The expression of the target genes was normalized using the reference gene (*β*-actin). The relative expression ratio of a target gene was calculated using the 2^−*ΔΔ*Ct^ method, where the mean value was obtained when normalized against the expression of the reference gene [49].

### 2.8. Statistics

A statistical package for social science (SPSS) (Ver. 16.0, Chicago, IL, USA) software was used to test normality (Shapiro–Wilk's test) and homogeneity of variance (Levene's test) of all data, then a one-way analysis of variance was conducted. A Tukey's post hoc test was used for multiple comparisons at *p*  < 0.05. In addition, polynomial orthogonal regression analyses were used to evaluate potential linear or quadratic impacts of dietary Tau on the physiological parameters. In all cases, *p*  < 0.05 was considered as significant.

## 3. Results

### 3.1. Morphometric and Maturation Indices

Survival and growth were not influence by dietary Tau inclusion (*p* > 0.05, Table 3). Supplementing diets with 4–8 g kg^−1^ Tau had shorter latency period from ESA to spawning and showed a quadratic trend. Moreover, shrimps fed the 4 g kg^−1^ Tau diet had a higher gonadosomatic index (GSI) in compared to control and the 8 g kg^−1^ Tau groups (*p*  < 0.05). Hepatopancreatic index was elevated by increasing dietary Tau up to 8 g kg^−1^ Tau compared to the control group (*p*  < 0.05) then decreased and showed a quadratic trend in response to dietary Tau level.

### 3.2. Antioxidant and Digestive Enzymes

**Figure 1:**
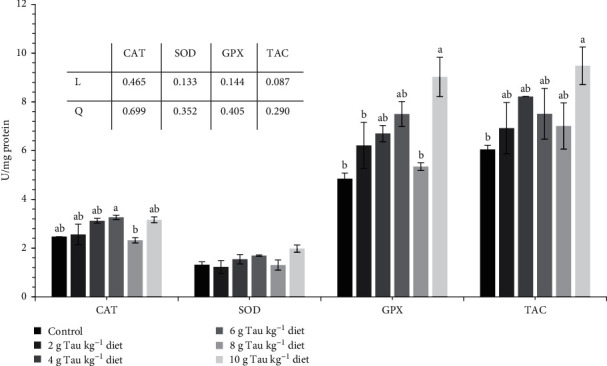
Antioxidant enzymes including CAT, SOD, GPx (U/mg protein), and TAC (µmol/g tissue) in hepatopancreas of *P. vannamei* female brooders (mean ± SEM, *n* = 3). Different superscripts on the bars denote differences (*p* < 0.05). Experimental groups fed experimental diets supplemented with different taurine levels including: 0 (control), 2, 4, 6, 8, and 10 g Tau kg^−1^ diet. CAT, catalase; GPx, glutathione peroxidase; SEM, standard error mean; SOD, superoxide dismutase; TAC; total antioxidant capacity.

**Figure 2:**
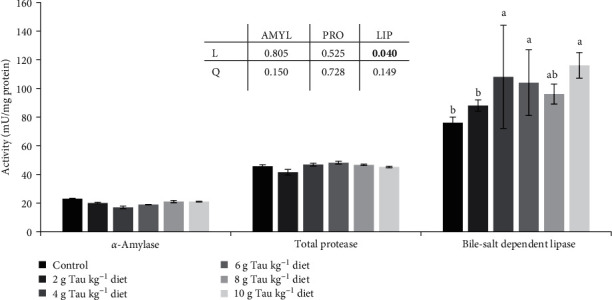
Digestive enzymes activity, including AMYL, total PRO, and bile-salt dependent LIP (mU/mg protein), in the gut f *P. vannamei* female brooders (mean ± SEM, *n* = 3). Different superscripts on the bars denote differences (*p* < 0.05). Experimental groups fed experimental diets supplemented with different taurine levels including: 0 (control), 2, 4, 6, 8, and 10 g Tau kg^−1^ diet. AMYL, *α*-amylase; LIP, lipase; PRO, protease; SEM, standard error mean. The significant *p* values (*p*  < 0.05) were shown in bold.

CAT activity in the 6 g kg^−1^ Tau-supplemented treatment was higher than 8 g kg^−1^ Tau-supplemented group (*p* < 0.05, Figure 1). The SOD activity did not change among groups (*p* > 0.05). GPx activity enhanced with increasing dietary Tau supplementation compared to the control except for those fed 8 g Tau kg^−1^ diet. TAC increased with increasing dietary Tau supplementation compared to the control, especially in Tau level of 10 g kg^−1^. In the present study, AMYL and total PRO activities were not affected by dietary Tau inclusion, but bile-salt dependent LIP activity increased by 4–10 g/kg Tau inclusion level and showed a positive linear trend (*p* < 0.05, Figure 2).

### 3.3. AA and FA Profiles of the Gonads

Regarding AA profile of the ovaries, except for Tau content, it was not significantly affected by dietary Tau supplementation and Tau level in the gonad were markedly increased with increasing Tau in the experimental diets (Table 4, *p* < 0.05). The amount of Tau and tyrosine showed both linear and quadratic trends in response to dietary Tau level.

The FA profile of the female shrimp ovaries showed that those fed 2 and 4 g kg^−1^ Tau-supplemented diets had lower total saturated FAs levels, mainly stearic acid (18 : 0), than control (*p* < 0.05, Table 5). With increasing the level of Tau in the experimental diet, the level of oleic acid was significantly increased. The highest and lowest arachidonic acid (ARA) levels were in those fed 2 and 8–10 g kg^−1^ Tau-supplemented diets, respectively, and its proportion showed negative linear trend in response to dietary Tau level. Docosahexaenoic acid (DHA) in shrimp fed 4 and 10 g Tau kg^−1^ diet was significantly higher than the control.

### 3.4. Plasma Biochemical Parameters

Plasma TP and Ca^+2^ increased in brooders fed Tau-supplemented diets (Table 6). The plasma CHOL level increased with increasing dietary Tau supplementation (*p* < 0.05). HDLs increased by supplementing diet with 4, 8, and 10 g Tau kg^−1^ compared to the control and showed a positive quadratic trend with increasing dietary Tau level. However, plasma TRIG did not show significant differences among groups.

### 3.5. Gene Expression

**Figure 3:**
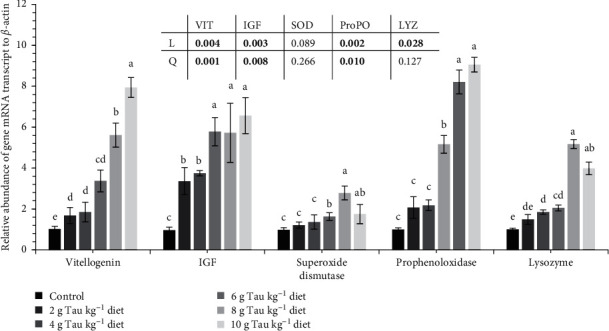
Relative abundance mRNA transcript of genes including: VIT, IGF, SOD, ProPO, and LYZ in *P. vannamei* female brooders hepatopancreas examined using real‐time quantitative RT‐qPCR. *β*-actin gene was used as an internal control to calibrate the cDNA template for all the samples (mean ± SEM, *n* = 3). Different superscripts on the bars denote differences (*p* < 0.05). Experimental groups fed experimental diets supplemented with different taurine levels including: 0 (control), 2, 4, 6, 8, and 10 g Tau kg^−1^ diet. IGF, insulin-like growth factor; LYZ, lysozyme; ProPO, prophenoloxidase; SEM, standard error mean; SOD, superoxide dismutase; VIT, vitellogenin. The significant *p* values (*p* < 0.05) were shown in bold.

Increasing dietary Tau upregulated the expression of *vtg*, *igf-II*, *sod*, *ProPO*, and *LYZ* genes in the hepatopancreas (*p* < 0.05, Figure 3). The highest and lowest *vtg*, *igf-II*, and *phenoloxidase* genes transcription levels were in shrimps fed with 10 g Tau kg^−1^ diet and the control group, respectively. The SOD and LYZ genes transcription levels were higher in shrimps fed 8 g Tau kg^−1^ diet than those fed 0–6 g Tau kg^−1^ diets.

## 4. Discussion

### 4.1. Growth and Reproductive Performance

The findings of the current research demonstrated that growth and survival of *P. vannamei* female brooders were not affected by dietary Tau inclusion suggesting during maturation, nutrients, and energy may channel to gonadogenesis and gametogenesis rather than somatic growth [31]. Previous studies in *P. vannamei* showed that dietary 0.4–0.8 g kg^−1^ Tau supplementation increased weight and protein utilization during grow-out stage (0.48 ± 0.0 g) [15]. Also, dietary Tau supplementation at 25 mg kg^−1^ enhanced the survival and moulting rate in *P. vannamei* during larval stage [50]. Furthermore, Shi et al. [20] reported that supplementing a diet in which large amount of FM was replaced by *Clostridium autoethanogenum* protein and soy protein concentrate with 4 g kg^−1^ Tau increased growth and feed utilization in *P. vannamei* (0.32 ± 0.00 g). Finally, Mai et al. [51] reported that dietary Tau increase shrimp adaptation response to low-temperature rearing condition and dietary Tau requirement at 28°C was 5.7–6.0 g kg^−1^, but at 20°C it was 5.6–6.6 g kg^−1^ in *P. vannamei* (1.59 ± 0.03 g) juveniles. Thus, based on the abovementioned studies, the optimal dietary Tau level is species-specific and even in the same species vary due to genetic, life stage, feed formulation, and experimental conditions among the other factors [52].

Broodstock nutrition has a vital role in producing high-quality gametes and offspring. AAs in free or peptide forms are critical nutrients as energy source, signaling molecules, and as precursors for bioactive compounds biosynthesis during the embryogenesis and early larval development [47]. Tau can protect germ cells from oxidative stress and increase spermatogenesis through increasing sex steroids effects in Japanese eel (*Anguilla japonica*) [27, 28]. In the present research, female brooders fed 4–8 g kg^−1^ Tau had higher HPI and shorter latency period after ESA indicating positive effects of dietary Tau on *P. vannamei* maturation condition. In addition, these findings suggested that dietary Tau supplementation contributed to the Tau pools in the ovaries and improved the sexual maturation in female brooders. Previous research in various fish species proved that Tau plays a pivotal role in reproductive performance and normal embryogenesis, and its dietary deficiency for brooder cannot be remedied by its administration during larviculture [23–26]. In this sense, Matsunari et al. [23] reported that supplementing diet with 10 g kg^−1^ increased oocyte growth, spawning, fertilization, and eggs hatching rates In yellowtail (*Seriola quinqueradiata*) that was coincided with an increase in total egg protein. Al-Feky, El-Sayed, and Ezzat [24] reported increased spawning frequency, fecundity, hatching rates, egg weight, egg protein content, and yolk-sac absorption times and heavier larvae at hatching when Nile tilapia (*Oreochromis niloticus*) brooders received Tau-supplemented feeds (10 g kg^−1^). In the case of *S. dorsalis*, 26.7 g kg^−1^ dietary Tau supplementation increased the proportion of floating and fertilized eggs, and the fecundity per brooder, larger yolk-sac volume in newly hatched, 53% more likely to survive to first-feeding than those from the control brooders [26]. However, regarding *S. dumerili* supplementing diet with 5–15 g kg^−1^ showed better results than Tau on reproductive performance. Nevertheless, dietary Tau supplementation at 3–11 g kg^−1^ improved *S. dumerili* fecundity compared to brooders fed a higher protein diet [25]. Finally, Guimarães et al. [53] reported that Tau did not affect growth and reproductive performance of zebrafish (*Danio rerio*) but it is important for normal lipid utilization and redox status.

### 4.2. Antioxidant Status

Tau by inhibiting lipid peroxidation, reducing apoptosis, and protecting the cell mitochondria from oxidation can increase antioxidant capacity of body [18]. Tau exert powerful antioxidant properties being. In particular, Tau is a potent scavenger of the ROS by increasing electron transport chain activity that prevents the electrons diversion from the respiratory chain to form superoxide anion [5]. In the present study, dietary Tau supplementation at 10 g kg^−1^ significantly enhanced hepatopancreas GPx and TAC and at 6 g kg^−1^ improved CAT activity suggesting Tau can modulate antioxidant defense status in *P. vannamei* female brooders. In this context, To and Liou [54] reported Tau (1 g kg^−1^) supplementation in diet in which 50% FM with soybean meal replacement could enhanced hemocytes GPx activity in *P. vannamei*. Moreover, Shi et al. [20] reported that CAT, GPx, SOD, and TAC decreased by dietary FM replacement with alternative protein sources, but their levels increased with dietary Tau (2–6 g kg^−1^) inclusion in *P. vanname* that coincided with the upregulation of GPx and SOD in the gut and GPx, SOD, and CAT in the hepatopancreas. Also, Dong et al. [18] reported that dietary 4–8 g kg^−1^ Tau can increase SOD, GPx, and TAC and reduce lipid peroxidation in *E. sinensis*. Also, Ding et al. [55] reported that dietary Tau (15 g kg^−1^) significantly enhanced TAC and reduced lipid peroxidation in freshwater prawn *Macrobrachium nipponense* after lead exposure stress. In juvenile ivory shell (*Babylonia areolata*), dietary Tau (10–20 g kg^−1^) also increased TAC and SOD activity and redcued lipid peroxidation [56]. In various fish species supplementing diet with Tau improved antioxidant capacity in *Totoaba macdonaldi* [57], common carp (*Cyprinus carpio*) [58], and red seabream (*Pagrus major*) [59].

### 4.3. Digestive Enzymes

Lipids digestion in the gut depends on LIPs and colipases, and bile acids as the main gut's biosurfactants are prerequisite for their activities [60, 61]. Tau participates in converting bile acids into bile salts process and promotes lipids and lipophilic substances absorption which is vital for lipid metabolism [9]. It has been reported that dietary Tau deficiency, especially in plant protein rich diets, can result in low digestive enzymes activity and low nutrients digestibility due to insufficient amounts of bile acids in the gallbladder and the gut [62, 63]. In the present study, bile-salt dependent LIP activity significantly increased by inclusion of 4–10 g Tau kg^−1^ diet, indicating positive effects of this AA on lipid metabolism in shrimp brooders. In this sense, Mai et al. [51] reported that supplementing diet with 2–8 g Tau kg^−1^ significantly improved digestive enzymes activity, such as LIP at optimum (28°C) and low (20°C) temperatures that associated with higher protein digestibility in *P. vannamei* juveniles. Supplementing diet with Tau significantly enhanced digestive enzyme activities in other farmed aquatic species such as meagre (*Argyrosomus regius*) [12], common dentex (*Dentex dentex*) [64], cobia larvae (*Rachycentron canadum*) [65], and Japanese amberjack (*Seriol quinqueradiata*) [66]. In addition, Sun et al. [56] reported that pepsin and LIP activities in both gut and hepatopancreas significantly increased by 10–20 g kg^−1^ Tau supplementation levels in Juvenile Ivory Shell.

### 4.4. AA and FA Profiles

EAA and protein retention rate are considered as the most reliable indices for dietary AA deficiencies [67]. In fact, an AA deficiency will adversely influence the biosynthesis of the protein molecules and also other AA availability [68]. In this research, the AA profile of gonads was unchanged except Tau that was gradually increased with the increment of Tau in diet that was similar to the results obtained in *S. dumerili* [25] and *S. dorsalis* [26] female brooders eggs. It should be mentioned that the EAA profile in the whole body is almost stable and is barely affected by dietary composition, because the protein biosynthesis is genetically determined [69, 70]. In contrast, Al-Feky, El-Sayed, and Ezzat [24] reported that supplementing diet with 10 g kg^−1^ Tau significantly enhanced lysine, arginine, isoleucine, and Tau in Nile tilapia eggs. Also, Dehghani et al. [13] reported that the EAA including leucine and lysine and also Tau gradually increased with increasing dietary Tau level in yellowfin seabream (*Acanthopagrus latus*) whole body. Marine shrimps have restricted capacity to synthesize *n*-3 long-chain polyunsaturated FAs (LC-PUFA) from their precursor the *α*-linolenic acid (18 : 3*n*−3) [71]; thus, their increment in shrimp ovaries may be due to other factors. In the present study, DHA level increased in shrimp fed Tau-supplemented diets, particularly those fed 10 g kg^−1^ Tau indicating positive effects of this nutrient on DHA retention and preservation in ovaries. Tau can improve lipid digestion and metabolism as indicated by the increment of bile-salt dependent LIP activity that may enhance the fat-soluble vitamins (e.g., vitamin E) absorption and consequently preserve DHA in the ovaries [9]. In this context, Cai et al. [72] reported that dietary Tau significantly increased the muscle *n*-3 PUFA in rice field eel (*Monopterus albus*) fed oxidized fish oil by increasing redox status and antioxidant capacity. Furthermore, Guo et al. [73] reported that the Tau intake improved the contention of DHA and reduced the *n*-6/*n*-3 ratio in mice serum compared to those fed oxidized oil mainly because of antioxidant effects of Tau. It should be mentioned that in the current study, an increase in DHA level was associated with a trivial reduction in ARA, particularly in those fed 8–10 g Tau kg^−1^ diets. In this context, Sun et al. [56] reported that dietary inclusion of 15 g Tau kg^−1^ increased 22 : 2*n*-6 and DHA proportions in juvenile ivory shell that coincided with a reduction in ARA proportion in this species. In addition, they showed that the increment of DHA proportion was associated with the increment of HDL in this species. These findings suggesting that dietary Tau by exerting positive effects on the antioxidant status and lipid metabolism can improve FA profile of gonads.

### 4.5. Plasma Biochemical Parameters

The evaluation of plasma TP and Ca^2+^ is an indirect methods to determine plasma vitellogenin (VIT) content because VIT is a calcium-rich phosphorylated protein [74]. In the current research, female shrimps fed Tau-supplemented diets had higher plasma TP and Ca^2+^ levels than the control group suggesting greater hemolymph VIT level in these groups. CHOL is the sole precursor of steroids that are synthesized at the inner mitochondrial membrane, in which the cytochrome P450 CHOL side chain cleavage enzyme converts CHOL to pregnenolone that enters the endoplasmic reticulum to produce steroids under further enzymatic reactions [75]. The low-density lipoproteins are involved in transferring lipids from the liver to peripheral tissues, meanwhile the HDL transports CHOL from peripheral tissues to the liver for catabolism [76]. In our research, shrimp brooders fed Tau-supplemented diets had the higher plasma CHOL and HDL contents suggesting an increase in FAs and CHOL mobilization from the hepatopancreas to the maturating ovaries. It seems that dietary Tau supplementation has increased shrimp brooders capacity for bile acid synthesis and CHOL absorption. In addition, these results were associated with increasing LIP activity, DHA retention, and enhanced hepatopancreatic VIT gene transcript level in brooders fed Tau-supplemented diets. In this snse, Sun et al. [56] reported that hepatopancreas HDL content showed quadratic response (first increased then decreased) with increasing Tau in diet in juvenile ivory Shell. Finally, supplementing plant protein-rich diets with Tau significantly enhanced plasma CHOL in *S. quinqueradiata* [61] and meagre [12] indicating Tau has an integral role in lipid metabolism in aquatic animals.

### 4.6. Gene Expression

There is scarce molecular information regarding nutritional regulation of Tau on vitellogenesis. The hepatopancreas has a main role in vitellogenesis in crustaceans such as *P. vannamei* [77]. The secondary vitellogenesis takes place in the oocyte, and VIT converts to vitellin that accumulates within the ovary to form the essential nutrients for the embryogenesis [78]. In the current research, the hepatopancreatic VIT gene transcription level increased in female brooders fed with the Tau-supplemented diet indicating vitellogenesis, and ovarian development was triggered Tau inclusion in diet.

The insulin-like growth factor (*igf*) is a network of peptides (*igf-I* and *igf-II*), receptors, and binding proteins that regulate mitosis, protein metabolism regulation, and somatic growth [79, 80]. In this regard, Sharawy et al. [47] reported that there was a positive correlation between *igf-II* gene expression and muscle growth in *P. vannamei* larvae. Also, previous studies in various fish species reported that supplementing diet with Tau significantly upregulated *igf*-I gene expression in rainbow trout [81], olive flounder [82], and red seabream [59] that associated with better growth performance. In addition, the *igf* family plays vital roles in steroidogenesis, follicle survival, ovulation, testis differentiation, and function in mammals [83]. In the current study, *igf-II* gene transcription upregulated in shrimp brooders fed Tau-supplemented diets, particularly those fed 6–10 g kg^−1^ Tau that was associated with upregulation of VIT gene transcription level in hepatopancreas suggesting Tau can modulate shrimp reproduction by modifying gonads development. As it was mentioned before, in the current research, supplementing diet with 10 g kg^−1^ increased hepatopancreas GPx and TAC and at 6 g kg^−1^ improved CAT activity that was associated with upregulation of *sod* gene expression in shrimp brooders fed 6–10 g kg^−1^ Tau. These findings suggest that dietary Tau can modulate antioxidant capacity by modulating antioxidant enzymes genes. In this sense, Guo et al. [73] reported that supplementing oxidized fish oil contained diet with 6–12 g kg^−1^ Tau markedly upregulated *sod* and *gpx* genes expression in the liver of mice that coincided with increasing SOD and GPx activities in the serum and liver.

Phenoloxidase catalyzes the oxidation of phenols to form quinines, which polymerized melanin. It induces hemocytes attraction and leads to melanization of pathogens and participates in cuticle sclerotization and wound healing [84]. LYZ is an important part of innate immune system that splits the peptidoglycan layer of Gram-positive bacteria and, as an opsonin trigger phagocytes to annihilate Gram-negative bacteria [85]. The results of the current study showed increasing mRNA transcript abundance of *ProPO* and *LYZ* in groups fed Tau-supplemented diets, especially those fed 6–10 g kg^−1^ Tau, suggesting the immunostimulatory effects of this supplement on *P. vannamei*. In this sense, Wang et al. [19] reported that 2.5 mg/mL Tau injection enhanced shrimp survival by more than 50% when challenged with *Vibrio parahaemolyticus* (PD-2), indicating that an optimal amount of Tau could enhance antibacterial response in penaeid shrimp. Moreover, Dong et al. [18] reported that the LYZ and phenoloxidase activities increased in the plasma and hepatopancreas of Chinese mitten crab fed 4–8 g Tau kg^−1^ diet. These findings indicating that dietary Tau can modulate innate immune responses of crustacean species.

## 5. Conclusion

In summary, in this research, supplementing diet with 4–8 g Tau kg^−1^ remarkably reduced latency period from eye stalk ablation to spawning in *P. vannamei* female brooders that were associated with the upregulation of *VIT* and *igf-II* genes expression, and the increment TP, Ca^+2^, CHOL, and HDL in plasma. In addition, bile-salt dependent LIP activity in the gut and DHA retention in ovaries of female brooders increased by dietary Tau inclusion. Finally, *ProPO*, *LYZ*, and *sod* genes upregulated in hepatopancreas of shrimp brooders fed diets containing 6–10 g kg^−1^ Tau suggesting that dietary Tau by modulating antioxidant capacity and immunocompetence can improve reproductive performance of *P. vannamei* female brooders.

## Figures and Tables

**Table 1:** Ingredients and proximate composition of experimental diets (dry matter).

Ingredients (g kg^−1^)^a^	Taurine (g kg^−1^)					
	0	2	4	6	8	10
Fishmeal^b^	360	360	360	360	360	360
Shrimp meal^b^	120	120	120	120	120	120
Wheat gluten meal^b^	120	120	120	120	120	120
Corn gluten meal^b^	120	120	120	120	120	120
Beef gelatin	25	25	25	25	25	25
Yeast	20	20	20	20	20	20
Fish oil^b^	26	26	26	26	26	26
Canola oil	26	26	26	26	26	26
Soy lecithin^c^	10	10	10	10	10	10
Cholesterol^d^	2	2	2	2	2	2
Vitamin and mineral premix^e^	23	23	23	23	23	23
Vitamin C^f^	3	3	3	3	3	3
Di-calcium phosphate^b^	15	15	15	15	15	15
Cellulose	10	8	6	4	2	0
Taurine^f^	0	2	4	6	8	10
Proximate analysis (g 100 g^−1^ of diet)						
Crude protein	49.2	49.5	49.3	49.8	50.2	50.1
Crude lipid	14	13.5	13.3	13.6	13.7	13.5
Crude ash	14.1	14	13.9	13.9	13.8	13.1
Moisture	8.5	8.8	8.2	8.8	8.3	8.6
Gross energy (MJ kg^−1^)^g^	19.6	19.5	19.5	19.6	19.7	19.7
Amino acid profile (g 100 g^−1^ of diet)						
Arginine	1.2	1.3	1.3	1.2	1.3	1.3
Histidine	1.4	1.4	1.6	1.5	1.5	1.6
Isoleucine	1.0	1.2	1.2	1.0	1.1	1.2
Leucine	4.0	3.8	3.7	3.9	3.8	3.9
Lysine	3.3	3.6	3.4	3.5	3.5	3.5
Methionine	1.1	1.0	0.9	1.0	0.9	0.9
Phenylalanine	2.7	2.4	2.5	2.7	2.5	2.5
Threonine	1.3	1.4	1.3	1.4	1.5	1.4
Valine	1.3	1.3	1.1	1.2	1.3	1.3
Aspartate	3.9	4.1	4.0	4.3	4.2	4.2
Glutamate	7.2	7.5	8.0	7.3	7.8	7.6
Serine	3.6	3.6	3.5	3.4	3.5	3.6
Glycine	5.3	5.5	5.8	5.1	5.5	5.6
Alanine	2.1	2.4	2.2	2.4	2.4	2.3
Proline	4.0	3.6	3.7	4.0	3.8	3.8
Cysteine	1.6	1.5	1.5	1.6	1.5	1.5
Cystine	0.5	0.6	0.6	0.5	0.6	0.5
Tryptophan	0.2	0.2	0.3	0.2	0.2	0.2
Tyrosine	1.1	1.0	1.1	1.0	0.9	1.1
Taurine	0.2	0.5	0.7	0.9	1.2	1.4

*Note:* The gross energy of the experimental feed was calculated on gross energy values of 23.6 kJ g^−1^ proteins, 39.5 kJ g^−1^ fat, and 17.2 kJ g^−1^ carbohydrates [29].

^a^Composition of ingredients (fish meal (520 g kg^−1^ crude protein, 180 g kg^−1^ crude lipid), shrimp meal [380 g kg^−1^ crude protein, 80 g kg^−1^ crude lipid], wheat gluten meal [500 g kg^−1^ kg crude protein, 30 g kg^−1^ crude lipid], corn gluten meal [520 g kg^−1^ crude protein, 30 g kg^−1^ crude lipid], beef, gelatin [850 g kg^−1^ crude protein, 42 g kg^−1^ crude lipid]).

^b^Havorash (Bushehr, Iran).

^c^Behpak Industrial Company, Behshahr, Mazandaran, Iran.

^d^Merck, Germany.

^e^Vitamin A, 5,000,000 IU; vitamin D3, 500,000 IU; vitamin E, 3000 mg; vitamin K3, 1500 mg; vitaminB1, 6000 mg; vitamin B2, 24,000 mg; vitamin B5, 52,000 mg; vitamin B6, 18,000 mg; vitamin B12, 60,000 mg; folic acid, 3000 mg; nicotinamide, 180,000 mg; antioxidant, 500 mg, copper, 3000 mg; zinc, 15,000 mg; manganese, 20,000 mg; Iron, 10,000 mg; potassiumiodate, 300 mg, career up to 1 kg, Damloran pharmaceutical company, Broujerd, Iran.

^f^Sumchun Pure Chemical, South Korea.

^g^Calculated on the basis of 24.3, 39.7, and 17.2 KJ/g of protein, lipid and carbohydrates, respectively.

**Table 2:** Primers used in the real-time quantitative RTPCR analyses.

Gene name	Accession number	Primers (forward/reverse) sequence	Amplicon	Efficiency	Reference
Vitellogenin	AY321153	F: GGTGTTGCTGTTGCTGCTGTGAA R: TTGACTAACTGAGATGAAG AGAAC	218	96	[46]

igf-II	XM02739466	F: CTCTGTACAGTCAGCCCAGC R: CACACCCAGTCAGTCCCAAG	220	98	[47]

sod	XM_027376216.1	F: AGCTTACATCTCCATCCTGG R: ATCTGAGGACTGACTGTGC	189	96	[48]

Prophenoloxidase	AY723296	F: CGGTGACAAAGTTCCTCTTC R: GCAGGTCGCCGTAGTAAG	122	97	[48]

Lysozyme	AY170126.2	F: TGT TCC GAT CTG ATG TCC R: GCT GTT GTA AGC CAC CC	121	98	[48]

*β*-Actin	AF300705.2	F: CCACGAGACCACCTACAAC R: AGCGAGGGCAGTGATTTC	142	97	[48]

**Table 3:** Morphometric and reproductive parameters of *P. vannamei* female brooders fed diets supplemented with nucleotide mixture.

	Taurine (g kg^−1^)						Regression	
	0	2	4	6	8	10	Linear	Quadratic
Morphometric parameters								
BW_i_ (g)	29.4 ± 0.9	29.4 ± 0.9	29.4 ± 0.9	29.4 ± 0.9	29.4 ± 0.9	29.4 ± 0.9	1.000	1.000
TL_i_ (cm)	16.5 ± 0.2	16.5 ± 0.2	16.5 ± 0.2	16.5 ± 0.2	16.5 ± 0.2	16.5 ± 0.2	1.000	1.000
BW_f_ (g)	34.4 ± 0.9	36.1 ± 0.8	35.4 ± 1.1	34.9 ± 1.0	37.5 ± 0.8	33.3 ± 3.3	0.900	0.577
TL_f_ (cm)	17.2 ± 0.1	17.2 ± 0.1	17.2 ± 0.2	17.1 ± 0.1	17.4 ± 0.1	17.2 ± 0.1	0.602	0.877
WG (%)	19.5 ± 2.8	23.7 ± 2.9	20.3 ± 3.9	18.8 ± 3.4	27.6 ± 2.9	19.7 ± 3.0	0.739	0.913
SGR (% BW day^−1^)	0.5 ± 0.1	0.65 ± 0.1	0.62 ± 0.1	0.55 ± 0.1	0.8 ± 0.1	0.58 ± 0.1	0.561	0.518
K (%)	0.7 ± 0.0	0.7 ± 0.0	0.7 ± 0.0	0.7 ± 0.0	0.7 ± 0.0	0.7 ± 0.0	1.000	1.000
Survival (%)	100 ± 0.0	100 ± 0.0	100 ± 0.0	100 ± 0.0	100 ± 0.0	100 ± 0.0	1.000	1.000
Reproductive parameters								
HPI (%)	2.9 ± 0.5^c^	3.4 ± 0.0^bc^	4.1 ± 0.2^ab^	4.6 ± 0.0^a^	4.7 ± 0.2^a^	4.0 ± 0.0^ab^	0.079	**0.019**
GSI (%)	2.7 ± 0.5^b^	4.3 ± 0.3^a^	3.2 ± 0.2^ab^	3.1 ± 0.4^ab^	2.6 ± 0.2^b^	3.0 ± 0.2^ab^	0.530	0.731
Absolute fecundity (×10^4^)	68.5 ± 21.5	56.1 ± 9.0	62.4 ± 5.3	45.8 ± 8.7	60.4 ± 5.3	72.3 ± 13.4	0.870	0.204
Eggs diameter (μm)	56.0 ± 4.3	49.0 ± 2.3	52.0 ± 2.9	46.0 ± 3.4	49.0 ± 3.5	45.0 ± 2.7	0.052	0.177
Latency period (days after eye stalk ablation to spawning)	12 ± 0.5^b^	11 ± 0.5^b^	5.0 ± 0.6^a^	4.0 ± 0.8^a^	6.0 ± 0.2^a^	12.0 ± 0.4^b^	0.662	**0.044**

*Note:* A different superscript in the same row denotes statistically significant differences (*p* < 0.05) (mean ± SEM, *n* = 3). WG = (BW_f_ − BW_i_)/BW_i_) × 100. SGR = [(ln BW_f_ − ln BW_i_)/*t*] × 100, where *t* is experimental period = 30 days. K = (BW_f_ (g)/TL_f_ (cm^3^) × 100. Survival = (number of specimens in each group remaining on day 30/initial number of specimens) × 100. HPI = (hepatopancreas weight (g)/BW_f_ (g)) × 100. GSI = (gonad weight (g)/BW_f_ (g)) × 100. The significant *p* values (*p* < 0.05) were shown in bold.

Abbreviations: BW_f_, final body weight; BW_i_, initial body weight; GSI, gonadosomatic index; HPI, hepatopancreatic index; K, Fulton's condition factor; SEM, standard error mean; SGR, specific growth rate; TL_f_, final total length; TL_i_, initial total length; WG, weight gain.

**Table 4:** Gonads amino acids profile (g 100 g^−1^ of wet weight) of *P. vannamei* fed taurine supplemented diets at the end of the feeding trial.

Amino acids profile	Taurine (g/kg)							Regression	
	0	2	4	6	8	10	Pooled SE	Linear	Quadratic
Arginine	0.2	0.2	0.3	0.2	0.3	0.2	0.0	0.414	0.742
Histidine	0.7	0.8	0.7	0.8	0.6	0.8	0.0	0.733	0.739
Isoleucine	2.5	2.4	2.5	2.6	2.5	2.6	0.2	0.172	0.436
Leucine	0.4	0.3	0.2	0.4	0.3	0.4	0.0	0.338	0.678
Lysine	0.9	1.0	1.1	1.0	1.0	1.1	0.1	0.172	0.380
Methionine	0.2	0.3	0.2	0.2	0.2	0.3	0.0	0.512	0.550
Phenylalanine	1.1	0.9	1.0	1.1	0.9	1.1	0.1	0.919	0.769
Threonine	0.6	0.7	0.8	0.7	0.6	0.7	0.0	0.894	0.619
Valine	0.8	1.0	1.1	0.8	0.9	0.9	0.1	0.931	0.748
Aspartate	1.9	2.0	2.2	1.8	2.1	2.1	0.1	0.527	0.843
Glutamate	2.9	3.0	2.8	3.1	2.9	3.1	0.2	0.381	0.669
Serine	1.0	0.9	1.0	1.1	1.0	1.1	0.1	0.172	0.436
Glycine	1.5	1.4	1.5	1.7	1.3	1.6	0.1	0.775	0.966
Alanine	0.6	0.8	0.7	0.6	0.5	0.7	0.0	0.626	0.904
Proline	2.5	2.3	2.7	2.5	2.3	2.4	0.2	0.637	0.791
Cysteine	0.1	0.2	0.1	0.1	0.1	0.2	0.0	0.694	0.735
Tryptophan	0.4	0.3	0.4	0.3	0.4	0.5	0.0	0.316	0.192
Tyrosine	0.5	0.6	0.7	0.7	0.8	0.8	0.1	**0.002**	**0.006**
Taurine	0.1^c^	0.3^b^	0.3^b^	0.5^b^	0.6^a^	0.8^a^	0.0	**0.001**	**0.005**

*Note:* A different superscript in the same row denotes statistically significant differences (*p* < 0.05) (mean ± SEM, *n* = 3). The significant *p* values (*p* < 0.05) were shown in bold.

Abbreviations: SE, standard error; SEM, standard error mean.

**Table 5:** Gonads fatty acid profile in *P. vannamei* female brooders fed different experimental diet (% total fatty acids).

Fatty acids	Taurine (g kg^−1^)						Regression	
	0	2	4	6	8	10	Linear	Quadratic
14 : 0	0.3 ± 0.0^bc^	0.2 ± 0.0^c^	0.4 ± 0.0^ab^	0.4 ± 0.0^ab^	0.4 ± 0.0^ab^	0.5 ± 0.0^a^	**0.042**	0.169
16 : 0	17.9 ± 0.1^ab^	17.1 ± 0.4^b^	17.5 ± 0.2^ab^	17.9 ± 0.2^ab^	18.7 ± 0.6^a^	17.7 ± 0.1^ab^	0.405	0.742
18 : 0	17.4 ± 0.6^a^	15.2 ± 0.5^b^	15.5 ± 0.4^b^	16.5 ± 0.1^ab^	17.6 ± 0.4^a^	15.9 ± 0.0^ab^	0.944	0.883
20 : 0	0.7 ± 0.1	0.6 ± 0.0	0.6 ± 0.0	0.7 ± 0.0	0.5 ± 0.1	0.6 ± 0.0	0.316	0.632
22 : 0	0.7 ± 0.1	0.5 ± 0.0	0.5 ± 0.0	0.4 ± 0.0	0.7 ± 0.1	0.4 ± 0.0	0.443	0.695
24 : 0	0.5 ± 0.0	0.5 ± 0.0	0.3 ± 0.1	0.6 ± 0.1	0.3 ± 0.0	0.3 ± 0.0	0.549	0.286
SFA	37.5 ± 0.3	34.1 ± 0.8	34.8 ± 0.3	36.5 ± 0.4	38.2 ± 1.5	35.4 ± 0.1	0.825	0.912
14 : 1*n−*5	0.2 ± 0.0	0.2 ± 0.0	0.2 ± 0.0	0.3 ± 0.0	0.2 ± 0.0	0.2 ± 0.0	0.805	0.655
16 : 1*n−*7	0.4 ± 0.1	0.6 ± 0.0	0.6 ± 0.0	0.6 ± 0.0	0.6 ± 0.0	0.6 ± 0.0	0.158	0.099
18 : 1*n−*7	2.1 ± 0.1	2.4 ± 0.1	2.4 ± 0.0	2.4 ± 0.1	2.4 ± 0.0	2.5 ± 0.1	0.066	0.133
18 : 1*n−*9	19.5 ± 0.6^b^	21.8 ± 0.3^a^	21.3 ± 0.2^a^	21.4 ± 0.2^a^	20.9 ± 0.9^ab^	21.8 ± 0.2^a^	0.256	0.394
20 : 1*n−*9	0.6 ± 0.1	0.8 ± 0.1	0.7 ± 0.0	0.7 ± 0.0	0.7 ± 0.0	0.8 ± 0.0	0.316	0.648
22 : 1*n−*9	0.2 ± 0.0	0.2 ± 0.0	0.2 ± 0.0	0.4 ± 0.0	0.2 ± 0.0	0.3 ± 0.1	0.374	0.675
MUFA	22.9 ± 0.6	25.9 ± 0.3	25.5 ± 0.3	25.6 ± 0.2	25.0 ± 0.2	26.2 ± 0.3	0.185	0.300
18 : 2*n−*6, LA	9.7 ± 0.0	10.1 ± 0.2	10.3 ± 0.1	10.2 ± 0.0	9.9 ± 0.1	10.3 ± 0.1	0.299	0.433
20 : 2*n−*6	0.9 ± 0.1	1.1 ± 0.1	0.9 ± 0.0	1.0 ± 0.0	0.9 ± 0.0	0.9 ± 0.0	0.538	0.683
20 : 4*n−*6, ARA	4.9 ± 0.4^ab^	5.5 ± 0.6^a^	4.6 ± 0.1^ab^	4.4 ± 0.0^ab^	3.9 ± 0.1^b^	4.0 ± 0.0^b^	**0.031**	0.139
*n*−6 PUFA	15.6 ± 0.5^ab^	16.8 ± 0.3^a^	15.8 ± 0.0^ab^	15.6 ± 0.1^ab^	14.7 ± 0.2^b^	15.3 ± 0.1^ab^	0.189	0.443
18 : 3*n−*3, LNA	1.2 ± 0.1	0.9 ± 0.0	1.0 ± 0.0	1.0 ± 0.1	1.0 ± 0.0	1.0 ± 0.0	0.457	0.416
20 : 3*n−*3	0.2 ± 0.1	0.3 ± 0.1	0.1 ± 0.0	0.2 ± 0.0	0.3 ± 0.0	0.1 ± 0.0	0.648	0.877
20 : 5*n−*3, EPA	9.1 ± 0.5	8.7 ± 0.3	9.3 ± 0.1	9.4 ± 0.1	8.0 ± 0.4	8.5 ± 0.1	0.313	0.523
22 : 6*n−*3, DHA	8.0 ± 0.3^b^	9.2 ± 0.2^ab^	9.8 ± 0.0^a^	8.8 ± 0.2^ab^	8.8 ± 0.0^ab^	10.4 ± 0.3^a^	0.188	0.480
*n−*3 PUFA	18.6 ± 0.3	19.1 ± 0.4	20.1 ± 0.1	19.4 ± 0.1	18.2 ± 0.9	20.0 ± 0.2	0.626	0.872
*n−*3/*n−*6	1.2 ± 0.0	1.1 ± 0.1	1.3 ± 0.2	1.2 ± 0.1	1.2 ± 0.0	1.3 ± 0.1	0.316	0.632
ARA/EPA	0.5 ± 0.0	0.6 ± 0.0	0.5 ± 0.0	0.5 ± 0.0	0.5 ± 0.0	0.5 ± 0.0	0.441	0.758
DHA/EPA	0.9 ± 0.1	1.1 ± 0.0	1.1 ± 0.1	0.9 ± 0.0	1.1 ± 0.0	1.2 ± 0.2	0.240	0.541

*Note:* A different superscript in the same row denotes statistically significant differences (*p* < 0.05) (mean ± SEM, *n* = 3). The significant *p* values (*p* < 0.05) were shown in bold.

Abbreviations: ARA, arachidonic acid; DHA, docosahexaenoic acid; EPA, eicosapentaenoic acid; LA, linoleic acid; LNA, linoleic acid; MUFA, monounsaturated fatty acids; *n−*3 PUFA, *n−*3 polyunsaturated fatty acids; *n−*6 PUFA, *n−*6 polyunsaturated fatty acids; SEM, standard error mean; SFA, saturated fatty acids.

**Table 6:** Plasma biochemical parameters of *P. vannamei* female brooders fed experimental diets (mean ± SEM, *n* = 3).

	Taurine (g kg^−1^)						Regression	
	0	2	4	6	8	10	Linear	Quadratic
Biochemical parameters								
Total protein (g dL^−1^)	5.6 ± 0.7^b^	8.8 ± 1.0^a^	8.3 ± 0.3^a^	7.6 ± 0.5^ab^	8.0 ± 0.2^a^	8.3 ± 0.1^a^	0.323	0.395
Cholesterol (mg dL^−1^)	26.4 ± 1.7^b^	30.3 ± 3.3^a^	33.4 ± 0.5^a^	28.0 ± 0.2^a^	33.9 ± 0.2^a^	33.8 ± 0.5^a^	0.123	0.348
Triglyceride (mg dL^−1^)	65.5 ± 18.6	44.5 ± 0.2	70.1 ± 20.1	97.2 ± 25.8	52.0 ± 9.7	75.4 ± 4.5	0.584	0.844
HDL (mg dL^−1^)	8.0 ± 0.4^b^	8.6 ± 0.6^ab^	9.7 ± 0.3^a^	8.4 ± 0.1^ab^	9.2 ± 0.1^a^	9.6 ± 0.1^a^	0.154	**0.007**
Calcium (mg dL^−1^)	10.7 ± 1.1^b^	13.7 ± 0.4^a^	12.7 ± 0.3^ab^	11.5 ± 0.4^ab^	13.5 ± 0.1^ab^	12.3 ± 0.2^ab^	0.582	0.681

*Note:* A different superscript in the same row denotes statistically significant differences (*p* < 0.05). The significant *p* values (*p* < 0.05) were shown in bold.

Abbreviation: SEM, standard error mean.

## Data Availability

The data are available from the corresponding author upon reasonable request.

## References

[1] FAO (2024). The State of World Fisheries and Aquaculture. *Blue Transformation in Action*.

[2] Ji D. W., Li F., Hu L. H. (2022). Metabolomics Analysis Revealed Biochemical Changes in Hepatopancreas and Ovary of *Litopenaeus vannamei* During Ovarian Maturation. *Aquaculture Reports*.

[3] Wouters R., Lavens P., Nieto J., Sorgeloos P. (2001). Penaeid Shrimp Broodstock Nutrition: An Updated Review on Research and Development. *Aquaculture*.

[4] Guillaume J., D’Abramo L. R., Conklin D. E., Akiyama D. M. (1997). Protein and Amino Acids. *Crustacean Nutrition*.

[5] Brown P. B. (1995). A Review of Nutritional Research With Crayfish. *Journal of Shellfish Research*.

[6] Qi C., Wang X., Han F. (2019). Arginine Supplementation Improves Growth, Antioxidant Capacity, Immunity and Disease Resistance of Juvenile Chinese Mitten Crab, *Eriocheir sinensis*. *Fish and Shellfish Immunology*.

[7] Balakrishna P., George S., Hatoum H., Mukherjee S. (2021). Serotonin Pathway in Cancer. *International Journal of Molecular Sciences*.

[8] Lefèvre P. L., Palin M.-F., Murphy B. D. (2011). Polyamines on the Reproductive Landscape. *Endocrine Reviews*.

[9] El-Sayed A. F. M. (2014). Is Dietary Taurine Supplementation Beneficial for Farmed Fish and Shrimp? A Comprehensive Review. *Reviews in Aquaculture*.

[10] Vessey D. A., Benfatto A. M., Zerweck E., Vestweber C. (1990). Purification and Characterization of the Enzymes of Bile Acid Conjugation From Fish Liver. *Comparative Biochemistry and Physiology Part B*.

[11] Ceccotti C., Al-Sulaivany B. S. A., Al-Habbib O. A. M., Saroglia M., Rimoldi S., Terova G. (2019). Protective Effect of Dietary Taurine From ROS Production in European Seabass Under Conditions of Forced Swimming. *Animals*.

[12] de Moura L. B., Diógenes A. F., Campelo D. A. V. (2019). Nutrient Digestibility, Digestive Enzymes Activity, Bile Drainage Alterations and Plasma Metabolites of Meagre (*Argyrosomus regius*) Feed High Plant Protein Diets Supplemented With Taurine and Methionine. *Aquaculture*.

[13] Dehghani R., Oujifard A., Mozanzadeh M. T., Morshedi V., Bagheri D. (2020). Effects of Dietary Taurine on Growth Performance, Antioxidant Status, Digestive Enzymes Activities and Skin Mucosal Immune Responses in Yellowfin Seabream, *Acanthopagrus latus*. *Aquaculture*.

[14] Kotzamanis Y., Tsironi T., Brezas A. (2020). High Taurine Supplementation in Plant Protein-Based Diets Improves Growth and Organoleptic Characteristics of European Seabass (*Dicentrarchus labrax*). *Scientific Reports*.

[15] Yue Y. R., Liu Y. J., Tian L. X. (2013). The Effect of Dietary Taurine Supplementation on Growth Performance, Feed Utilization and Taurine Contents in Tissues of Juvenile White Shrimp (*Litopenaeus vannamei*, Boone, 1931) Fed With Low-Fishmeal Diets. *Aquaculture Research*.

[16] Richard L. F., Vachot C., Surget A., Rigolet V., Kaushik S. J., Geurden I. (2011). The Effect of Choline and Cystine on the Utilisation of Methionine for Protein Accretion, Remethylation and Trans-Sulfuration in Juvenile Shrimp *Penaeus monodon*. *British Journal of Nutrition*.

[17] Zhou M., Wu Z., Liang R., Gu N. (2017). Effects of Dietary Taurine, Carnitine and Cholesterol Supplementation on Growth Performance and Immunological Status of *Litopenaeus vannamei* Under Cold Exposure. *Aquaculture Research*.

[18] Dong J., Cheng R., Yang Y. (2018). Effects of Dietary Taurine on Growth, Non-Specific Immunity, Anti-Oxidative Properties and Gut Immunity in the Chinese Mitten Crab *Eriocheir sinensis*. *Fish & Shellfish Immunology*.

[19] Wang Z., Aweya J. J., Yao D. (2022). Taurine Metabolism Is Modulated in Vibrio-Infected *Penaeus vann*amei to Shape Shrimp Antibacterial Response and Survival. *Microbiome*.

[20] Shi M., Yao X., Qu K., Liu Y., Tan B., Xie S. (2023). Effects of Taurine Supplementation in Low Fishmeal Diet on Growth, Immunity and Intestinal Health of *Litopenaeus vannamei*. *Aquaculture Reports*.

[21] Chen K., Xiong W., Liu W., Cao X., Chi C., Jiang G. (2024). Effects of Dietary Methionine and Taurine Interaction on Growth Performance, the Profiles of Amino Acids, and Protein Metabolism of Chinese Mitten Crab, *Eriocheir sinensis*. *Aquaculture Reports*.

[22] Shiau S. Y., Chou B. S. (1994). Grass shrimp, *Penaeus monodon*, Growth as Influenced by Dietary Taurine Supplementation. *Comparative Biochemistry and Physiology—Part A: Physiology*.

[23] Matsunari H., Hamada K., Mushiake K., Takeuchi T. (2006). Effects of Taurine Levels in Broodstock Diet on Reproductive Performance of Yellowtail *Seriola quinqueradiata*. *Fisheries Science*.

[24] Al-Feky S. S. A., El-Sayed A.-F. M., Ezzat A. A. (2016). Dietary Taurine Improves Reproductive Performance of Nile Tilapia (*Oreochromis niloticus*) Broodstock. *Aquaculture Nutrition*.

[25] Sarih S., Djellata A., Roo J. (2018). Effects of Increased Protein, Histidine and Taurine Dietary Levels on Egg Quality of Greater Amberjack (*Seriola dumerili*) Risso, 1810). *Aquaculture*.

[26] Salze G. P., Davis D. A., Stuart K., Drawbridge M. (2019). Effect of Dietary Taurine in the Performance of Broodstock and Larvae of California Yellowtail *Seriola Dorsalis*. *Aquaculture*.

[27] Higuchi M., Celino F. T., Miura C. (2012). *The Synthesis and Role of Taurine in the Eel Spermatogenesis, in: Interdisciplinary Studies on Environmental Chemistry—Environmental Pollution and Ecotoxicology*.

[28] Higuchi M., Celino F. T., Tamai A., Miura C., Miura T. (2012). The Synthesis and Role of Taurine in the Japanese Eel Testis. *Amino Acids*.

[29] National Research Council (1993). *Nutrient Requirements of Fish National 514*.

[30] Maneii K., Oujifard A., Ghasemi A., Mozanzadeh M. T. (2019). Reproductive Performance and Vitellogenin mRNA Transcript Abundance in the Hepatopancreas of Female *Litopenaeus vannamei* Fed Diets With Different Soy Lecithin Content. *Animal Reproduction Science*.

[31] Arshadi A., Yavari V., Oujifard A., Mousavi S. M., Gisbert E., Mozanzadeh M. T. (2018). Dietary Nucleotide Mixture Effects on Reproductive and Performance, Ovary Fatty Acid Profile and Biochemical Parameters of Female Pacific Shrimp *Litopenaeus vannamei*. *Aquaculture Nutrition*.

[32] Alday-Sanz V. (2010). *The Shrimp Book*.

[33] Simpson A. C. (1951). The Fecundity of the Plaice. *Fisheries Investigation London*.

[34] Folch J., Lees N., Sloane-Stanley G. H. (1957). A Simple Method for the Isolation and Purification of Total Lipids From Animal Tissues. *Journal of Biological Chemistry*.

[35] Christie W. W., Christie W. W. (1993). Preparation of Ester Derivatives of Fatty Acids for Chromatographic Analysis. *Advances in Lipid Methodology*.

[36] Agh N., Jasour M. S., Noori F. (2014). Potential Development of Value-Added Fishery Product in Underutilized and Commercial Fish Species: Comparative Study of Lipid Quality Indicators. *Journal of the American Oil Chemists’ Society*.

[37] Lindroth P., Mopper K. (1979). High Performance Liquid Chromatographic Determination of Subpicomole Amounts of Amino Acids by Precolumn Fluorescence Derivatization With o-Phthaldialdehyde. *Analytical Chemistry*.

[38] Aebi H. (1984). Catalase in Vitro. *Methods in Enzymology*.

[39] Kono Y. (1978). Generation of Superoxide Radical During Autoxidation of Hydroxylamine and an Assay for Superoxide Dismutase. *Archives of Biochemistry and Biophysics*.

[40] Günzler A., Flohé L., Greenwald R. A. (1985). Glutathione Peroxidase. *CRC Handbook of Methods for Oxygen Radical Research 1*.

[41] Chong A. S. C., Hashim R., Chow-Yang L., Ali A. B. (2002). Partial Characterization and Activities of Proteases From the Digestive Tract of Discus Fish (*Symphysodon aequifasciata*). *Aquaculture*.

[42] Bernfeld P. (1955). Amylases, *α* and *β*. *Methods in Enzymology*.

[43] Iijima N., Tanaka S., Ota Y. (1998). Purification and Characterization of Bile Salt-Activated Lipase From the Hepatopancreas of Red Sea Bream, *Pagrus Major*. *Fish Physiology and Biochemistry*.

[44] Folin O., Ciocalteau V. (1929). Enzymatic Assay of Protease Using Casein as a Substrate. *Journal of Biological Chemistry*.

[45] Bradford M. M. (1976). A Rapid and Sensitive Method for the Quantitation of Microgram Quantities of Protein Utilizing the Principle of Protein-Dye Binding. *Analytical Biochemistry*.

[46] Raviv S., Parnes S., Segall C., Davis C., Sagi A. (2006). Complete Sequence of *Litopenaeus vannamei* (Crustacea: Decapoda) Vitellogenin cDNA and Its Expression in Endocrinologically Induced Sub-Adult Females. *General and Comparative Endocrinology*.

[47] Sharawy Z. Z., Ashour M., Abbas E. (2020). Effects of Dietary Marine Microalgae, Tetraselmis Suecica, on Production, Gene Expression, Protein Markers and Bacterial Count of Pacific White Shrimp *Litopenaeus vannamei*. *Aquaculture Research*.

[48] Abbaszadeh A., Mozanzadeh M. T., Qasemi A., Oujifard A., Bahabadi M. Nafsi (2022). Efects of the Addition of *Calanopia elliptica*, *Artemia franciscana*, and *Brachionus rotundiformis* in a Nursery Biofoc System on Water Quality, Growth, Gut Morphology, Health Indices, and Transcriptional Response of Immune and Antioxidant-Related Genes in *Penaeus Vanname*. *Aquaculture International*.

[49] Pfaffl M. W. (2001). A New Mathematical Model for Relative Quantification in Real-Time RT-PCR. *Nucleic Acids Research*.

[50] Mayasari N. (2005). The Effect of Taurine to Speed up Molting and Increase Physical Endurance From Vanname Shrimp Larva *Litopenaeus vannamei* (Boone). *World Aquaculture 2005*.

[51] Mai H., Li Y., Song Z. (2025). Effect of Taurine on Growth and Immune Response of Pacific White Shrimp (*Litopenaeus vannamei*) Cultured at Different Temperatures. *Aquaculture*.

[52] Zhu R., Wu X.-Q., Zhao X.-Y. (2022). Taurine Can Improve Intestinal Function and Integrity in Juvenile *Rhynchocypris lagowskii Dybowski* Fed High-Dose Glycinin. *Fish & Shellfish Immunology*.

[53] Guimarães I. G., Skjærven K., Moren M., Espe M., Hamre K. (2018). Dietary Taurine Supplementation in Plant Protein Based Diets Do Not Affect Growth and Reproductive Performance of Zebrafish. *Aquaculture Research*.

[54] To V.-A., Liou C.-H. (2021). Taurine Supplementation Enhances the Replacement Level of Fishmeal by Soybean Concentrate in Diets of Juvenile Pacific White Shrimp (*Litopenaeus vannamei* Boone, 1931). *Aquaculture Research*.

[55] Ding Z., Kong Y., Qi C., Liu Y., Zhang Y., Ye J. (2021). The Alleviative Effects of Taurine Supplementation on Growth, Antioxidant Enzyme Activities, Hepatopancreas Morphology and mRNA Expression of Heat Shock Proteins in Freshwater Prawn *Macrobrachium nipponense* (De Haan) Exposed to Dietary Lead Stress. *Aquaculture Nutrition*.

[56] Sun Y., Du X., Yang Y., Wang A., Gu Z., Liu C. (2023). Dietary Taurine Intake Affects the Growth Performance, Lipid Composition, and Antioxidant Defense of Juvenile Ivory Shell (*Babylonia areolata*). *Animals*.

[57] Bañuelos-Vargas I., López L. M., Pérez-Jiménez A., Peres H. (2014). Effect of Fishmeal Replacement by Soy Protein Concentrate With Taurine Supplementation on Hepatic Intermediary Metabolism and Antioxidant Status of Totoaba Juveniles (*Totoaba macdonaldi*). *Comparative Biochemistry and Physiology Part B: Biochemistry and Molecular Biology*.

[58] Abdel-Tawwab M., Monier M. N. (2018). Stimulatory Effect of Dietary Taurine on Growth Performance, Digestive Enzymes Activity, Antioxidant Capacity, and Tolerance of Common Carp, *Cyprinus carpio* L., Fry to Salinity Stress. *Fish Physiology and Biochemistry*.

[59] Gunathilaka B. E., Kim M.-G., Lee C., Lee K.-J., Lee K.-J. (2019). Effects of Taurine Supplementation in Low Fish Meal Diets for Red Seabream (Pagrus Major) in Low Water Temperature Season. *Fisheries and Aquatic Sciences*.

[60] Tocher D. R. (2010). Metabolism and Functions of Lipids and Fatty Acids in Teleost Fish. *Reviews in Fisheries Science*.

[61] Bakke A. M., Glover C., Krogdahl Å., Grosell A. P. F. M., Colin J. B. (2011). Feeding, Digestion and Absorption of Nutrients. *Fish Physiology*.

[62] Nguyen H. P., Khaoian P., Fukada H., Nakamori T., Furuta H., Masumoto T. (2011). Effects of Different Soybean Proteins on Lipid Digestion and Growth of Yellowtail *Seriola quinqueradiata*. *Fisheries Science*.

[63] Nguyen P. H., Khaoian P., Furutani T., Nagano J., Fukada H., Masumoto T. (2011). Effects of Alcohol Extract from Soybean Meal on Pancreatic Digestive Enzyme and Bile Acid Secretion in Yellowtail *Seriola quinqueradiata*. *Aquaculture Science*.

[64] Chatzifotis S., Polemitou I., Divanach P., Antonopoulou E. (2008). Effect of Dietary Taurine Supplementation on Growth Performance and Bile Salt Activated Lipase Activity of Common Dentex, *Dentex dentex*, Fed a Fish Meal/Soy Protein Concentrate-Based Diet. *Aquaculture*.

[65] Salze G., McLean E., Craig S. R. (2012). Dietary Taurine Enhances Growth and Digestiveenzyme Activities in Larval Cobia. *Aquaculture*.

[66] Nguyen H. P., Khaoian P., Fukada H., Suzuki N., Masumoto T. (2015). Masumoto Feeding Fermented Soybean Meal Diet Supplemented With Taurine to Yellowtail Seriola Quinqueradiata Affects Growth Performance and Lipid Digestion. *Aquaculture Research*.

[67] Bulbul M., Kader M. A., Koshio S., Ishikawa M., Yokoyama S. (2014). Effect of Replacing Fishmeal With Canola Meal on Growth and Nutrient Utilization in Kuruma Shrimp *Marsupenaeus japonicus* (Bate). *Aquaculture Research*.

[68] Harper A. E., Benevenga N. J., Wohlhueter R. M. (1970). Effects of Ingestion of Disproportionate Amounts of Amino Acids. *Physiological Reviews*.

[69] Alam M., Yaniharto D., Sumule O., Ishikawa M., Koshio S. (2005). Assessment of Reference Dietary Amino Acid Pattern for Juvenile Red Sea Bream, *Pagrus Major*. *Aquaculture International*.

[70] Whiteman K. W., Gatlin D. M. (2005). Evaluation of Crystalline Amino Acid Test Diets Including pH Adjustment With Red Drum (*Sciaenops ocellatus*) and Hybrid Striped Bass (*Morone chrysops× Morone saxatilis*). *Aquaculture*.

[71] Gonzalez-Felix M. L., Gatlin D. M., Lawrence A. L., Perez-Velazquez M. (2002). Effect of Dietary Phospholipid on Essential Fatty Acid Requirements and Tissue Lipid Composition of *Litopenaeus vannamei* Juveniles. *Aquaculture*.

[72] Cai M., Chu W., Wang J. (2022). Intervention of Taurine on Fatty Acid Profiles, Oxidative Injury and Autophagy Status in the Muscle of Rice Field Eel (*Monopterus albus*) Fed Oxidized Fish Oil. *Aquaculture*.

[73] Guo Q., Zhang L., Yin Y. (2022). Taurine Attenuates Oxidized Fish Oil-Induced Oxidative Stress and Lipid Metabolism Disorder in Mice. *Antioxidants*.

[74] Verslycke T., Vandenbergh G. F., Versonnen B., Arijs K., Janssen C. R. (2002). Induction of Vitellogenesis in 17a-Ethinylestradiolexposed Rainbow Trout (*Oncorhynchus mykiss*): A Method Comparison. *Comparative Biochemistry and Physiology Part C*.

[75] Hall P. F. (1985). Role of Cytochromes P-450 in the Biosynthesis of Steroid Hormones. *Vitamins & Hormones*.

[76] Bai F., Niu X., Wang X., Ye J. (2021). Growth Performance, Biochemical Composition and Expression of Lipid Metabolism Related Genes in Groupers (*Epinephelus coioides*) Are Altered by Dietary Taurine. *Aquaculture Nutrition*.

[77] Fatima H., Ayub Z., Ali S. A., Siddiqui G. (2013). Biochemical Composition of the Hemolymph, Hepatopancreas, Ovary, and Muscle during Ovarian Maturation in the Penaeid Shrimps *Fenneropenaeus merguiensis* and *F. penicillatus*. *Turkish Journal of Zoology*.

[78] Abdu U., Davis C., Khalaila I., Sagi A. (2002). The Vitellogenin cDNA of *Cherax quadricarinatus* Encodes a Lipoprotein With Calcium Binding Ability, and Its Expression Is Induced Following the Removal of the Androgenic Gland in a Sexually Plastic System. *General and Comparative Endocrinology*.

[79] Giustina A., Mazziotti G., Canalis E. (2008). Growth Hormone, Insulin-Like Growth Factors, and the Skeleton. *Endocrine Reviews*.

[80] Jung H., Lyons R. E., Hurwood D. A., Mather P. B. (2013). Genes and Growth Performance in Crustacean Species: A Review of Relevant Genomic Studies in Crustaceans and Other Taxa. *Reviews in Aquaculture*.

[81] Gaylord T. G., Teague A. M., Barrows F. T. (2006). Taurine Supplementation of All-Plant Protein Diets for Rainbow Trout (*Oncorhynchus mykiss*). *Journal of World Aquaculture Society*.

[82] Kim J. M., Malintha G. H. T., Gunathilaka G. L. B. E. (2017). Taurine Supplementation in Diet for Olive Flounder at Low Water Temperature. *Fisheries and Aquatic Sciences*.

[83] Neirijnck Y., Papaioannou M. D., Nef S. (2019). The Insulin/IGF System in Mammalian Sexual Development and Reproduction. *International Journal of Molecular Sciences*.

[84] Amparyup P., Charoensapsri W., Tassanakajon A., Saurabh S., Sahoo P. K. (2013). Prophenoloxidase System and Its Role in Shrimp Immune Responses Against Major Pathogens. *Fish and Shellfsh Immunology*.

[85] Saurabh S., Sahoo P. K. (2008). Lysozyme: An Important Defense Molecule of Fish Innate Immune System. *Aquaculture Research*.

[86] Finn R. N., Fyhn H. J. (2010). Requirement for Amino Acids in Ontogeny of Fish. *Aquaculture Research*.

